# Outcomes of sugar reduction policies, United Kingdom of Great Britain and Northern Ireland 

**DOI:** 10.2471/BLT.23.291013

**Published:** 2024-03-27

**Authors:** Kawther M Hashem, Hattie E Burt, Mhairi K Brown, Graham A MacGregor

**Affiliations:** a Wolfson Institute of Population Health, Barts and The London School of Medicine and Dentistry, Queen Mary University of London, Charterhouse Square, London, EC1M 6BQ, England.

## Abstract

Poor diets are the major cause of death and disease globally, driving high levels of obesity and noncommunicable diseases. Cheap, heavily marketed, ultra-processed, energy-dense and nutrient-poor food and drinks that are high in fat, sugar and salt play a major role. The high-sugar content of these products leads to consumption levels much higher than recommended. The World Health Organization recommends that sugar intake should be reduced to just 5% of energy intake by using fiscal policies and food and drink reformulation strategies. Over the previous decade, the government of the United Kingdom of Great Britain and Northern Ireland has implemented several policies aimed at reducing sugar intake. We compare the soft drinks industry levy and the sugar reduction programme, examining how differences in policy design and process may have influenced the outcomes. Success has been mixed: the mandatory levy achieved a reduction in total sugar sales of 34.3%, and the voluntary reduction programme only achieved a 3.5% reduction in sugar levels of key contributors to sugar intake (despite a target of 20%). Both policies can be improved to enhance their impact, for example, by increasing the levy and reducing the sugar content threshold in the soft drinks industry levy, and by setting more stringent subcategory specific targets in the sugar reduction programme. We also recommend that policy-makers should consider applying a similar levy to other discretionary products that are key contributors to sugar intake. Both approaches provide valuable learnings for future policy in the United Kingdom and globally.

## Résumé

La malnutrition est l'une des principales causes de décès et de pathologies dans le monde, entraînant des taux élevés d'obésité et un grand nombre de maladies non transmissibles. Massivement commercialisés, les aliments et boissons bon marché, ultra-transformés, riches en énergie et pauvres en nutriments, à forte teneur en graisse, en sucre et en sel jouent un rôle majeur. La quantité de sucre contenue dans ces produits engendre une consommation qui dépasse largement les recommandations en la matière. L'Organisation mondiale de la Santé conseille de réduire la proportion de sucre afin que ce dernier ne représente plus que 5% de l'apport énergétique grâce à des politiques fiscales et des stratégies de révision de la composition des aliments et des boissons. Au cours des dix dernières années, le gouvernement du Royaume-Uni de Grande-Bretagne et d'Irlande du Nord a adopté plusieurs politiques visant à réduire la consommation de sucre. Dans le présent document, nous comparons la taxe sur l'industrie des sodas avec le programme de réduction du sucre, en examinant comment les différences de conception et de mise en œuvre des politiques pourraient avoir influencé les résultats. Le succès s'est révélé mitigé: la taxe obligatoire a permis de faire chuter le total des ventes de sucre de 34,3%, alors que le programme de baisse volontaire n'a pas permis de faire diminuer ce taux de plus de 3,5% chez les acteurs clés de l'apport en sucre (bien loin des 20% ciblés). Les deux politiques peuvent être améliorées pour renforcer leur impact, par exemple en augmentant la taxe et en réduisant la teneur en sucre maximale applicable à l'industrie des sodas, mais aussi en définissant des objectifs spécifiques plus stricts dans les sous-catégories du programme de réduction du sucre. Nous encourageons en outre les responsables politiques à instaurer une taxe similaire sur d'autres produits non essentiels qui contribuent eux aussi à la consommation de sucre. Les deux approches fournissent des renseignements précieux pour de futures mesures au Royaume-Uni et partout dans le monde.

## Resumen

Las dietas inadecuadas son la principal causa de muerte y enfermedad en todo el mundo. Además, impulsan altos niveles de obesidad y enfermedades no transmisibles. Los alimentos y las bebidas baratos, muy comercializados, ultraprocesados, hipercalóricos y pobres en nutrientes, con un alto contenido en grasas, azúcar y sal, desempeñan una función importante. El alto contenido en azúcar de estos productos conduce a niveles de consumo muy superiores a los recomendados. La Organización Mundial de la Salud recomienda reducir el consumo de azúcar a solo el 5% de la ingesta energética mediante políticas fiscales y estrategias de reformulación de alimentos y bebidas. En la última década, el gobierno del Reino Unido de Gran Bretaña e Irlanda del Norte ha aplicado varias políticas encaminadas a reducir la ingesta de azúcar. Comparamos el impuesto del sector de las bebidas no alcohólicas y el programa de reducción del azúcar, examinando cómo las diferencias en el diseño y el proceso de las políticas pueden haber influido en los resultados. El éxito ha sido desigual: el impuesto obligatorio logró una reducción de las ventas totales de azúcar del 34,3%, y el programa de reducción voluntaria solo consiguió una reducción del 3,5% en los niveles de azúcar de los principales contribuyentes a la ingesta de azúcar (a pesar de un objetivo del 20%). Se pueden mejorar ambas políticas para aumentar su impacto, por ejemplo, aumentando el impuesto y reduciendo el umbral de contenido de azúcar en el impuesto del sector de las bebidas no alcohólicas, y estableciendo objetivos específicos por subcategorías más estrictos en el programa de reducción de azúcar. También recomendamos a los responsables de formular las políticas que estudien la posibilidad de aplicar un impuesto similar a otros productos discrecionales que contribuyen decisivamente a la ingesta de azúcar. Ambos enfoques aportan valiosas enseñanzas para las futuras políticas del Reino Unido y del resto del mundo.

## ملخص

إن الأنظمة الغذائية الرديئة هي السبب الرئيسي للوفاة والأمراض على مستوى العالم، مما يؤدي إلى مستويات مرتفعة من السمنة والأمراض غير المعدية. إن الأطعمة والمشروبات الرخيصة، والتي تحظى بحملات تسويقية مكثفة، وتخضع لعمليات معالجة متصاعدة، وتزخر بمحتوى مركز من الطاقة، وبها محتويات فقيرة بالمغذيات، وذات نسبة عالية من الدهون، والسكر، والملح، هي أغذية تلعب دورًا رئيسيًا في هذه الأنظمة الرديئة. يؤدي المحتوى العالي من السكر في هذه المنتجات إلى مستويات استهلاك أعلى بكثير من الموصى بها. توصي منظمة الصحة العالمية بتخفيض نسبة السكر إلى %5 فقط من استهلاك الطاقة، وذلك باتباع السياسات المالية، واستراتيجيات إعادة صياغة الأطعمة والمشروبات. على مدى العقد الماضي، قامت حكومة المملكة المتحدة لبريطانيا العظمى وأيرلندا الشمالية بتنفيذ العديد من السياسات الهادفة إلى الحد من استهلاك السكر. قمنا بمقارنة ضريبة صناعة المشروبات الغازية وبرنامج خفض السكر، ودراسة كيفية تأثير الاختلافات في تصميم السياسات والعمليات على النتائج. وكانت نتائج النجاح مختلطة: فقد حققت الضريبة الإجبارية انخفاضًا في إجمالي مبيعات السكر بنسبة %34.3، بينما لم يحقق برنامج الخفض الطوعي سوى انخفاض بنسبة %3.5 في مستويات السكر لدى المنتجات الرئيسية الأخرى التي تسهم في استهلاك السكر (على الرغم من أن الهدف كان %20). ويمكن تحسين كلتا السياستين لتعزيز تأثيرهما، فعلى سبيل المثال، يتم القيام بذلك من خلال زيادة الضريبة، وخفض حد محتوى السكر في ضريبة صناعة المشروبات الغازية، ومن خلال وضع أهداف محددة أكثر صرامة لفئات فرعية في برنامج خفض السكر. نوصي أيضًا بأن واضعي السياسات يجب أن يضعوا في اعتبارهم فرض ضريبة مشابهة على المنتجات التقديرية الأخرى التي تعد مساهمة بشكل رئيسي في استهلاك السكر. ويقدم كلا الأسلوبين دروسًا مستفادة ذات قيمة للسياسة المستقبلية في المملكة المتحدة والعالم.

## 摘要

饮食不合理是全球的主要致死和患病原因，还造成了肥胖和非传染性疾病的高发性。其中，廉价、大批量销售、超加工、高能量和低营养的高脂肪、高糖和高盐食品和饮料的影响非常大。这些产品的高含糖量导致人们的摄入量远远超过了推荐量。世界卫生组织建议，通过财政政策和食品饮料配方调整策略，将糖的摄入量减少到仅占能量摄入量的 5%。在过去十年中，大不列颠及北爱尔兰联合王国政府实施了几项旨在减少糖摄入量的政策。我们比较了软饮料行业的征税情况和减糖计划，以研究政策在设计方面和实施过程中的差异会对成果造成怎样的影响。成果好坏参半：强制性征税使糖总销量减少了 34.3%，而自愿减糖计划仅使高糖摄入人群的糖摄入量减少了 3.5%（尽管目标是 20%）。可以改进这两项政策以增强其影响，例如，通过增加征税和降低软饮料行业征税中的含糖量门槛，以及在减糖计划中设定更严格的特定子类别目标。我们还建议，政策制定者应该考虑对其他会大大增加糖摄入量的弹性消费产品征收类似的税。这两种方法都为英国和全球未来的政策制定提供了宝贵的经验。

## Резюме

Неправильное питание является основной причиной смертности и возникновения заболеваний во всем мире и приводит к высокому уровню ожирения и развитию неинфекционных заболеваний. Главную роль играют дешевые, активно продаваемые, энергетически насыщенные и бедные питательными веществами продукты глубокой переработки и напитки с высоким содержанием жира, сахара и соли. Высокое содержание сахара в этих продуктах приводит к тому, что их потребление значительно превышает рекомендуемые нормы. Всемирная организация здравоохранения рекомендует сократить потребление сахара до 5% от общего количества потребляемой энергии путем использования фискальной политики и стратегий изменения состава продуктов питания и напитков. За последнее десятилетие Правительством Соединенного Королевства Великобритании и Северной Ирландии был реализован ряд мероприятий, направленных на снижение потребления сахара. В статье проводится сравнение налогового сбора с производителей газированных напитков и программы по снижению потребления сахара, а также изучается, как различия в разработке политики и процессе ее реализации могли повлиять на результаты. Успех оказался неоднозначным: в результате введения обязательного налогового сбора общий объем продаж сахара сократился на 34,3%, в то время как в рамках программы добровольного сокращения удалось добиться снижения уровня сахара в основных продуктах, способствующих его потреблению, лишь на 3,5% (при целевом показателе 20%). Обе политики могут быть усовершенствованы для усиления их воздействия, например, путем повышения налогового сбора и снижения порогового содержания сахара в налоге на газированные напитки, а также путем установления более строгих целей по конкретным подкатегориям в программе по снижению потребления сахара. Лицам, ответственным за принятие решений, также рекомендуется рассмотреть возможность введения аналогичного налогового сбора на другие, выбранные по собственному усмотрению продукты, которые являются основными причинами потребления сахара. Оба подхода позволяют извлечь ценные уроки для формирования дальнейшей политики в Соединенном Королевстве Великобритании и Северной Ирландии, а также во всем мире.

## Introduction

Excessive sugar consumption, increasing the risk of weight gain, is associated with an increased risk of type 2 diabetes, certain noncommunicable diseases and 13 types of cancer.^1^^,^^2^ There is also a causal relationship between sugar intake and tooth decay.^3^ In the United Kingdom of Great Britain and Northern Ireland, dental caries is the most frequently cited reason for the admission of children (age 6–10 years) to hospital for tooth extractions performed under general anaesthetic from 2016 to 2020.^4^

One of the main contributing factors to excessive sugar intake is the unhealthy food system that exists in many countries.^5^ The global food system is dominated by multinational corporations who exercise significant power over the options available to consumers:^6^ the largest 100 food and drink manufacturers account for 77% of all packaged food sold.^7^ These companies produce and market cheap, highly processed, energy-dense and nutrient-poor food and drinks that are high in sugar.^8^^–^^10^ The addition of sugar is largely driven by an excessive supply of sugar. In the United Kingdom, for example, three times the amount of sugar recommended for consumption at a population level is supplied to the market, facilitated by the liberalization of the European sugar market in the mid-2000s and a strong domestic industry.^11^

Because of the negative effect of high sugar intake on health, in 2015 the World Health Organization (WHO) issued a strong recommendation to reduce sugar intake to less than 10% of energy intake. WHO also issued the conditional recommendation that, for optimum health benefits, sugar intake be reduced to just 5% of energy intake.^1^ The European Food Safety Authority stated that sugar intake should be “as low as possible, in line with a nutritionally adequate diet.”^3^ As a percentage of energy intake, sugar intake in adults ranges from 6.9% in Portugal to 18.1% in Austria.^12^ Sugar consumption is especially high among children (age 4–10 years) and adolescents (age 11–18 years), averaging over 12% of energy intake in the United Kingdom.^13^ WHO recommends that countries reduce the sugar intake of their populations by using food and nutrition labelling, consumer education, restricted marketing of food and non-alcoholic beverages, fiscal policies, and food and drink reformulation strategies.^1^

## Sugar reduction policies

Evidence shows that sugary drinks taxes are associated with higher prices and often lower sales, which can contribute towards reducing sugar consumption.^14^ Taxes on sugary drinks have been implemented in more than 45 countries and several local jurisdictions.^14^

Reformulation involves companies improving the nutrition profile of products, by gradually reducing harmful elements such as excess sugar.^15^ The key advantage of reformulation is that it removes the individual burden of behaviour change or financial considerations from consumers, who can continue to buy the same products that become healthier over time. The well-proven approach of a government-led reformulation programme, and one that has demonstrated to be effective for salt, is to implement a set of average and/or maximum limits for the target nutrient in relevant food and drink product categories.^16^ The United Kingdom was one of the first countries to implement this approach for salt reduction in the early 2000s, setting targets for the 80 categories of food contributing most salt to the diet. This implementation resulted in reductions of salt content in relevant foods of 20–40%, alongside an overall reduction of 1.4 g salt per day in population intake.^16^

Identified as a leader in sugar reduction policy by WHO,^17^ the United Kingdom has developed and implemented several policies over the last decade aimed at reducing population sugar intake. The Scientific Advisory Committee on Nutrition independently reached the same conclusion as WHO, and recommended the adoption of the population target of reducing energy intake from sugar to 5%.^1^ This recommendation was based on government analysis that found that such action could prevent 3500 deaths and 173 000 dental caries cases annually, saving the National Health Service 396 million pounds sterling (£) each year.^18^

**Fig. 1:**
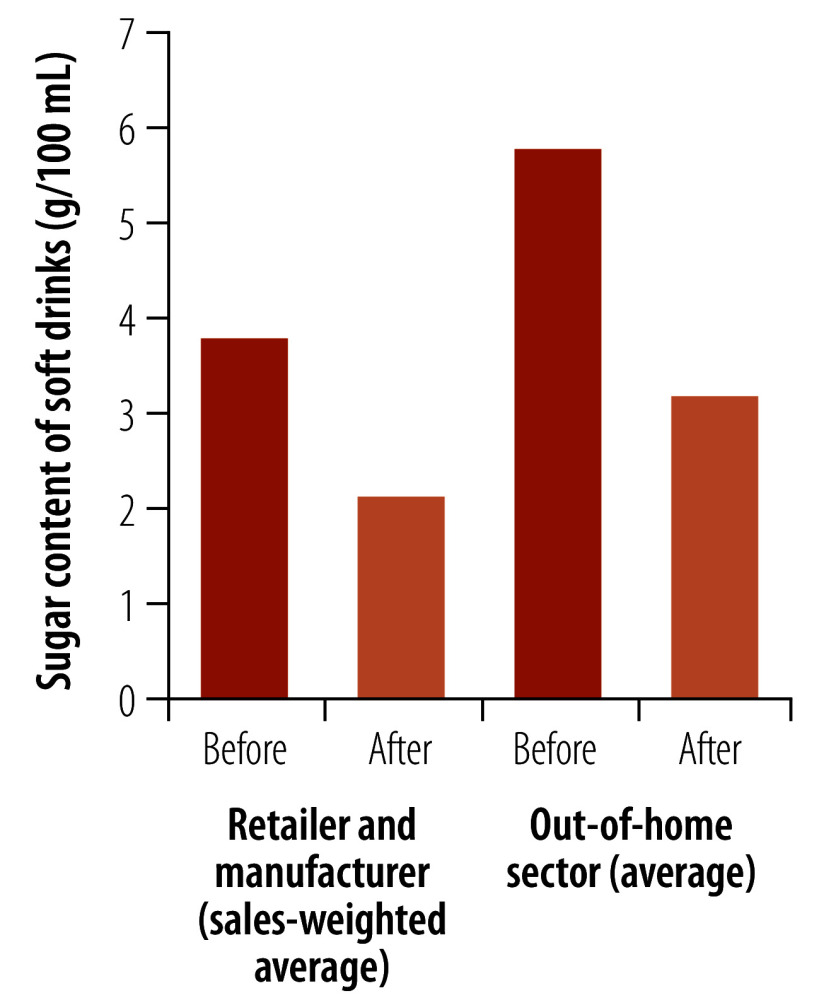
Average sugar content of beverages before and after the introduction of the soft drinks industry levy, United Kingdom

Source: United Kingdom Office for Health Improvements and Disparities.^19^

**Fig. 2:**
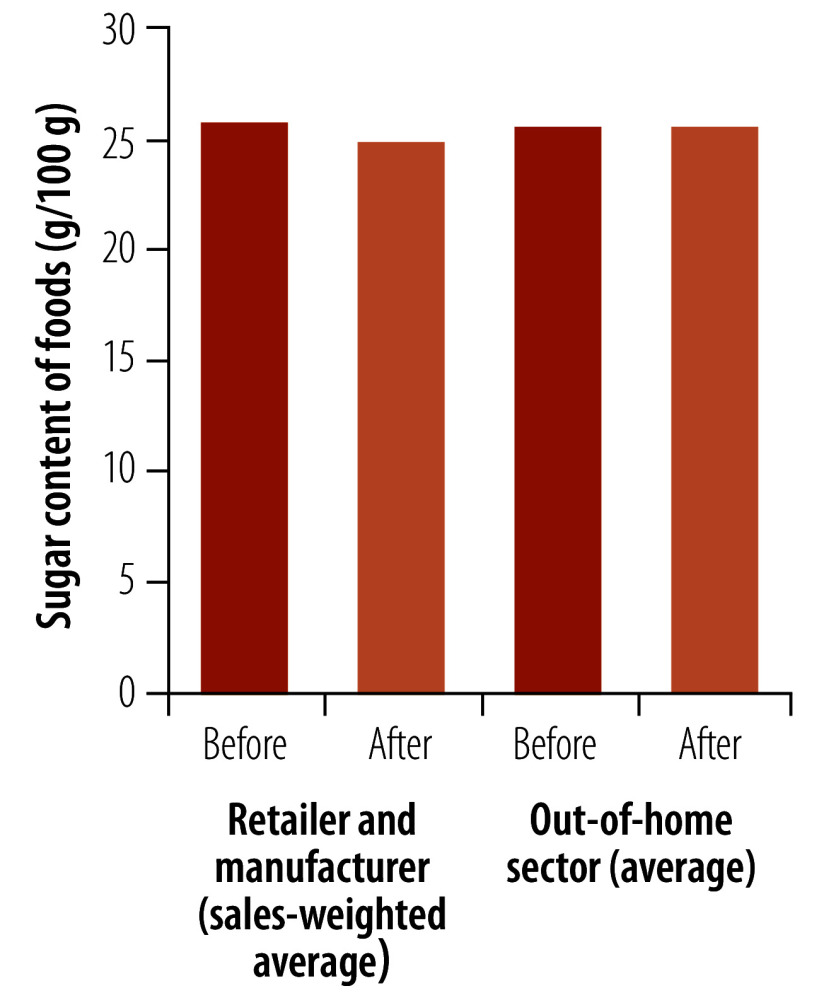
Average sugar content of food products before and after the introduction of the voluntary sugar reduction programme, United Kingdom

Source: United Kingdom Office for Health Improvements and Disparities.^19^

We compare two critical policy actions: the soft drinks industry levy and the sugar reduction programme (Fig. 1; Fig. 2; and Table 1). We examine how differences in policy design and process, which were mainly focused on encouraging reformulation of products high in sugar, may have influenced outcomes. We also highlight potential improvements to these policies that can provide valuable learning for other countries.

**Table 1:** Summary of policy details and improvement recommendations of the soft drinks industry levy and sugar reduction programme, United Kingdom

Feature	Soft drinks industry levy	Sugar reduction programme
Type	Fiscal measure imposed on the production of soft drinks with added sugars	Voluntary programme targeting companies to reduce their total sugar sales through reformulation, reducing portion size and shifting sales to lower-sugar options
Categories in scope	All soft drinks with 5 g of sugar per 100 mL in its ready-to-drink or diluted form, added during production or anything (other than fruit juice, vegetable juice and milk) that contains sugar (e.g. honey); includes drinks with a content of ≤ 1.2% alcohol by volume or less; since 2023, levy also applies to flavour concentrate drinks	All high-sugar categories such as: breakfast cereals; yoghurts; biscuits; cakes; morning goods; desserts and dessert toppings/sauces; ice cream, lollies and sorbets; chocolate and sweet confectionery; sweet spreads and sauces; chocolate spread; peanut butter; and fruit spreads. Since 2017, all drinks categories not included in the soft drinks levy such as milk-based drinks, juice and juice-based drinks, and fermented yoghurt drinks
Thresholds/target	Drinks with a total sugar content of 5–8 or ≥ 8 g/100 mL incur a levy of £0.18 or £0.24 per litre	To reduce sugar by at least 20% by 2020, including a 5% reduction in the first year of the programme by sectors of the food and drinks industry, across a range of products that contribute most to the sugar intakes of children
Monitoring process	Monitored with published annual reports; tax authority monitors revenue generated from registered companies	Monitored with published annual reports
Total sugar reduction	34.3% reduction in total sugar sales, from 135 391 tonnes in 2015 to 89 019 tonnes in 2020	3.5% reduction in total sugar during 2015–2020
Options available to companies	Reformulation, but can reduce portion size and sell less volume to retrieve some of the cost of the levy on the company	Reformulation, reducing portion size and shifting sales through marketing tactics
Improvement recommendations	Increase the levy, such that companies pay more levy to maintain formulation; reduce the sugar thresholds to encourage further reformulation; include milk-based and juice-based drinks; update nutrition labelling to include amount of non-sugar sweeteners	Divide existing product categories into more specific targets; make the more specific targets mandatory; include stricter guidelines for baby and toddler foods; trial a levy in the style of soft drinks levy on a poorly performing food category (e.g. chocolate confectionery; update nutrition labelling to include 'free sugars' over 'total sugars')

£: pounds sterling.

## Soft drinks industry levy

### Development

In 2008–2012, soft drinks (defined as high-sugar drinks) accounted for 17% of the free sugar intake in children aged 4–10 years, 30% in adolescents aged 11–18 years and 16% in adults aged 19–64 years in the United Kingdom.^20^ The concept of introducing taxes on soft drinks was initially proposed in recommendations made to the government by the independent agency Public Health England in their 2015 sugar reduction report.^18^ This recommendation suggested the introduction of a tax, already implemented in many other countries, in the form of a minimum price increase of 10–20% on high-sugar products including soft drinks. The introduction of a tax was encouraged by health organizations in the United Kingdom, as well as the announcement of one of its biggest retailers to reduce the sugar content of their own soft drinks by 5% each year from 2015.^21^

The soft drinks industry levy was formally announced in the 2016 budget, when it was stated that the government would introduce an industry levy in 2018 on manufacturers, packagers and importers of soft drinks, excluding small companies.^22^ The levy varies according to the sugar content of the drink, from no levy for drinks with less than 5 g sugar/100 mL; £0.18/L for drinks with 5–8 g sugar/100 mL; and £0.24/L for drinks with more than 8 g sugar/100 mL. 

The first tax of this kind, the levy aimed to directly incentivize the reformulation of products. The government promised to allocate the funds generated by the levy towards improving children’s health, including investment in children’s breakfast clubs and school sports facilities and activities. Ring-fencing the raised funds in this way assisted in the proposed levy becoming accepted policy, despite opposition from the food industry.^23^

The Office for Health Improvement and Disparities (previously Public Health England) monitored progress of the levy on behalf of the tax authority of the United Kingdom (His Majesty’s Revenue and Customs), publishing reports in 2018, 2019, 2020 and 2022. All relevant companies are required to comply with the levy by registering with the tax authority, submitting a levy return every quarter and paying any levy due. Interest, penalties and, in some cases, criminal charges are liable if the process is not followed.^24^

### Successes and failures

The tiered structure of the levy meant that many companies were incentivized to reformulate, despite some initial consumer backlash. As a result, there was a 34.3% reduction in total sugar sales from soft drinks from 135 391 tonnes in 2015 to 89 019 tonnes in 2020.^19^ During this period, the sales-weighted average sugar content of soft drinks subject to the levy decreased from 3.8 to 2.1 g/100 mL from 2015 to 2020 (by 44.7%, although quoted as 46.0%) for retailer own-brand and manufacturer-branded products.^19^ There was a reduction in the out-of-home sector (i.e. those businesses that sell food and drink consumed at locations other than at home) from 5.8 g/100mL in 2017 to 3.2 g/100 mL in 2020 (by 44.8%, although quoted as 44.3%), reported as a simple average because of data limitations.^19^ Because the levy was mandatory, no company was disadvantaged for taking action that their competitors did not.^25^

Recent evidence also suggests that the levy may have prevented over 5000 cases of obesity in girls aged 10–11 years, and may have reduced the number of adolescents requiring hospital tooth extractions by 12.1% (95% confidence interval: 7.2–17.0).^26^^,^^27^

Despite large reductions in sugar content, the volume of sales of soft drinks increased by 21.3% from 2015 to 2020 driven by a large shift towards lower sugar products; industry fears that the levy would lead to a loss of sales and profit were therefore allayed.^19^ Interviews with industry representatives have revealed that companies used surrogate marketing to ensure that sales of high-sugar drinks continued to increase.^28^

### Recommendations

To encourage more companies to reformulate, the value of the levy could be increased. To encourage further reformulation among the companies that have already reformulated their products, while also addressing some of the increase in sales due to aggressive marketing, we recommend reducing the sugar thresholds. 

Because reformulation to reduce sugar content (especially in the high-sugar drinks category) has resulted in an increase in the use of non-sugar sweeteners, further sugar reductions will probably mean further increases in the use of non-sugar sweeteners. Although previously seen as a viable way to reduce sugar content,^29^ this stance is shifting as new evidence emerges. WHO has reported that policy-makers should avoid the use of non-sugar sweeteners for long-term weight management or chronic disease prevention, and has recently issued a guideline on this topic.^30^ This recommendation raises the question of how to design policies that not only reduce sugar content but also discourage its replacement with non-sugar sweeteners. Some countries have started to incorporate non-sugar sweeteners into sugar reduction policies, for example: soft drink levies apply to high-sugar and non-sugar-sweetened drinks in France, Saudi Arabia, United Arab Emirates and the United States of America; the public health product tax in Hungary has recently expanded to apply to the content of non-sugar sweeteners as well as sugar;^31^ and the mandatory front-of-pack warning label system in Mexico includes information on non-sugar sweetener content. The use of non-sugar sweeteners is currently not monitored in the United Kingdom; we recommend a reduction in use as well as requiring companies to state the amount in products on nutrition labels.

There is potential to expand high-sugar drinks taxes to a wider range of products; for example, milk-based drinks and fruit juices are currently excluded from the levy, but are consumed in similar volumes as high-sugar drinks. Specific attention should also be given to alcoholic drinks, a major contributor to sugar intake but largely excluded from sugar reduction policies. In 2020, a survey of ready-to-drink alcoholic beverages found that an average serving contained 20 g sugar, something that consumers are largely unaware of because of a lack of nutrition labelling.^32^

Finally, although the funds raised by the levy were earmarked for public health initiatives, low revenue is indicative of a successful reformulation scheme; this contradiction is something that policy-makers must be aware of.

## Sugar reduction programme

### Development

In the previously mentioned 2015 sugar reduction report,^18^ Public Health England also recommended that the government introduce a “broad, structured and transparently monitored programme of gradual sugar reduction in everyday food and drink products, combined with reductions in portion size.” In the 2016 childhood obesity plan,^33^ the government announced the introduction of a “broad, structured sugar reduction programme to remove sugar from the products children eat most,” including breakfast cereals, yoghurts, cakes, biscuits, sweets and chocolate, reducing calories where possible.^29^

After extensive engagement and discussion with 40 organizations who represented all sectors of the food industry, nongovernmental organizations and other government departments, Public Health England developed the details of the reduction programme in 2016–2017. Learnings from previous government policies, such as the salt reduction programme, were also considered.^34^

The programme was aimed at manufacturers, retailers and out-of-home businesses with a significant market share in the top 10 food categories (breakfast cereals; yoghurts and fromage frais; biscuits; cakes; morning goods; puddings; ice cream, lollies and sorbets; chocolate confectionery; sweet confectionery; and sweet spreads and sauces) that contribute to the sugar intake of children.^29^ The government agency determined the food categories via the National Diet and Nutrition Survey, and calculated market share using data purchased from Kantar Worldpanel, representative of United Kingdom population intake. Following engagement with key stakeholders, who were also invited to provide written feedback, the agency published the aims of the programme: a 20% target reduction in sugar between 2015 (baseline) and 2020, including a 5% target in the first year. Companies could achieve this via three mechanisms: reformulation, portion size reductions and shifting sales to healthier alternatives. To aid progress, the agency also published baseline sales-weighted average sugar content by product category and the corresponding sugar content that would represent a 20% reduction.^29^ The key success marker was a reduction in the sales-weighted average sugar content per 100 g, calculated by weighting sugar levels of individual products by their volume sales. A popular product with high-sugar content drives the sales-weighted average upwards, whereas a popular product with low-sugar content drives it down.^29^

Analysis suggested that if the programme targets were met, average sugar consumption would fall by 1000–3600 g per person per year,^35^ with a resulting reduction in calorie intake capable of halting weight gain at a population level.^36^ The increased and healthier workforce could grow the United Kingdom’s economic output by £2.2–5.7 billion, and save the National Health Service £1.6–4.1 billion and the social care system £1.9–4.8 billion.^35^

Public Health England was tasked with managing the programme, using annual reports to track industry progress. A commitment was made that ministers would consider alternative levers if enough progress was not made.^29^ The agency published interim progress reports in 2018, 2019 and 2020; following its dissolution in 2020, the Office of Health Improvement and Disparities took responsibility and published the final report in December 2022. The report provided a detailed assessment, by food category and business, of progress over the 4-year programme towards the 20% reduction goal.^19^

### Successes and failures 

The 20% reduction goal was quickly criticized by industry,^37^ who claimed it was unachievable; however, there were some supportive voices among industry members.^38^

Overall, the programme achieved a 3.5% reduction in sales-weighted average sugar levels in retailer (6.3%) and manufacturer (1%) branded products between 2015 and 2020.^19^ Encouraging reductions were seen in the categories of breakfast cereals (by 14.9%) and yoghurts and fromage frais (by 13.5%), in which the programme demonstrated the potential of a reformulation approach.^19^ There were large differences between what the various companies achieved, with an average of 18 percentage points between the best and worst performers in each product category.

However, despite the reductions seen, there was a 7.1% increase (51 986 tonnes) in total sugar sales from foods.^19^^,^^38^ Out-of-home companies such as fast-food outlets and restaurant chains only achieved a 0.2% reduction in average sugar content. Calculating a meaningful measurement of sugar-weighted sales was not possible as the available data did not match purchases with nutrition information at the product level. At a company level, data were scarce and were not broken down for each food category.

The programme was criticized for being simplistic in its design, with a blanket 20% target reduction in sugar across all categories. This design did not follow the proven model of other successful reformulation programmes, such as the salt reduction programme, with specific and data-driven targets for each category.^16^ Given the three available methods to achieve the 20% reduction target, companies had the flexibility to easily overcome technical challenges to meet this goal. However, as the programme was voluntary, less responsible companies chose to make no or little changes, with many lower-sugar versions of high-sugar products being introduced to the market.^39^

The outcome of the 20% reduction was determined by sales-weighted average sugar content, but this outcome was not only affected by the sugar content of products; as sales changed, perhaps because of sales promotions, the absolute sugar content required to achieve the 20% target changed. This sales-weighting method also encouraged some companies to develop products with 30% less sugar, apply this claim to packaging, and market them alongside full-sugar product lines to bring consumers to the category. This strategy may explain some of the increase in total sugar sales. Furthermore, some companies were then unwilling to reformulate their main full-sugar products as the 30%-less-sugar variants would lose their status. The design of the programme may therefore have made sugar reduction a short-term marketing opportunity for some companies, rather than a long-term strategic aim to reduce sugar intake across the population.

### Recommendations

Although the programme covered a reasonably comprehensive range of products, it could be strengthened by dividing existing product categories into more specific targets (similar to the salt reduction programme targets), and expanding to include all top contributors to sugar intake for both adults and children. For example, baby and toddler foods should have been included in the programme with more stricter guidelines. Despite being a crucial period in a child’s development, proposed policies have failed to cover products marketed for the early years. This failure has resulted in excessively sugary products: recent surveys of commercial baby and toddler products found that 37% of sweet snacks would receive a red (high) label for sugars (> 22.5 g of total sugars per 100 g) according to United Kingdom colour-coded labelling,^32^^,^^40^ and that breakfast items contained up to 14.5 g sugar/pouch.^41^

Sugar reduction targets must be mandatory; industry will only prioritize voluntary targets if aligned with commercial interests. The chief executives of the United Kingdom’s major food retailers have stated that legislation is needed, emphasizing that “they need a level playing field if they are to start making their products healthier, otherwise the competition will simply move in and undercut them.”^35^ The successful salt targets imposed by the South African government demonstrate the power of mandatory targets to incentivize widespread reformulation to improve population health. In 2016, legislation was implemented limiting salt levels in key contributors of salt, such as bread, and the resulting reformulation led to an estimated reduction in population salt intake of 1.2 g/day by 2018.^42^

## Discussion

The United Kingdom experience in sugar reduction policy has demonstrated mixed success with some clear reductions in sugar content (Fig. 1 and Fig. 2), but the impact of the interventions may have been moderated by an increase in sales. Because these policies are implemented within an environment of an ever-changing market and economy, interventions must be flexible enough to be able to adapt to how companies mitigate the impact of these policies on their products.^28^

Both the soft drinks industry levy and the sugar reduction programme are examples of upstream measures that reach the whole population, without the need for individual action. These measures have been shown to achieve better health impacts than downstream measures, which attempt to change individual consumer behaviour.^43^ Both policies can be improved to continue their success and mitigate their failures, such as increasing the levy and reducing the sugar content threshold in the soft drinks industry levy, and setting more stringent subcategory specific targets in the sugar reduction programme (Table 1). 

The poorest performing category in the sugar reduction programme was chocolate confectionery, with only a 0.9% reduction over 4 years. Given the proven success of the soft drinks industry levy at incentivizing reformulation, we therefore recommend that policy-makers consider applying a similar levy to other discretionary products that are key contributors to sugar intake. In fact, one of the criticisms of the levy from the soft drinks industry was that their product was unfairly targeted and that a broader range of products should have been included.^25^ Hungary’s public health product tax, which applies to all pre-packaged products with added sugar, was successful in incentivizing reformulation^44^ and has recently been strengthened to include a new double-rate tax for particularly sweet products.^30^

Ultimately, we recommend a comprehensive programme of complementary policies to reduce sugar intake, incorporating those measures recommended by WHO such as: updating food and nutrition labelling legislation to help consumers better understand the amount of free sugars in products; and restrictions on advertising of high-sugar products, currently being delayed in the United Kingdom as a result of industry pressure.^45^
